# Tooth Movement Efficacy of Retraction Spring Made of a New Low Elastic Modulus Material, Gum Metal, Evaluated by the Finite Element Method

**DOI:** 10.3390/ma14112934

**Published:** 2021-05-29

**Authors:** Naohiko Tamaya, Jun Kawamura, Yoshinobu Yanagi

**Affiliations:** 1 Department of Dental Informatics, Graduate School of Medicine, Dentistry and Pharmaceutical Sciences, Okayama University, Okayama 700-8525, Japan; tama1026@msn.com (N.T.); ya7@okayama-u.ac.jp (Y.Y.); 2 Private Practice in Tamaya Orthodontic Office, Fukui 910-0851, Japan; 3 Private Practice in Kawamura Dental Office, Gifu 502-0847, Japan

**Keywords:** orthodontics, space closure, retraction spring, gum metal, finite element method

## Abstract

The aim of this study was to evaluate the tooth movement efficacy of retraction springs made of a new β-titanium alloy, “gum metal”, which has a low Young’s modulus and nonlinear super elasticity. Using double loop springs incorporated into an archwire made of gum metal (GUM) and titanium molybdenum alloy (TMA), the maxillary anterior teeth were moved distally to close an extraction space. The long-term movements were simulated by the finite element method. Its procedure was constructed of two steps, with the first step being the calculation of the initial tooth movement produced by elastic deformation of the periodontal ligament, and in the second step, the alveolar socket was moved by the initial tooth movement. By repeating these steps, the tooth moved by accumulating the initial tooth movement. The number of repeating calculations was equivalent to an elapsed time. In the GUM and TMA springs, the anterior teeth firstly tipped lingually, and then became upright. As a result of these movements, the canine could move bodily. The amount of space closure in GUM spring was 1.5 times that in TMA spring. The initial tipping angle of the canine in the GUM spring was larger than that in the TMA spring. The number of repeating calculations required for the bodily movement in the GUM spring was about two times that in the TMA spring. It was predicted that the speed of space closure in the GUM spring was smaller than that in the TMA spring.

## 1. Introduction

In orthodontic treatment, when there is not enough space to arrange the dentition, the first premolars are extracted and the anterior teeth are moved distally. Two methods have been utilized to move the teeth.

One method is sliding mechanics, in which an archwire fixed to the anterior teeth slides along the bracket slot of the posterior teeth. Translational or bodily movement of the anterior teeth can be easily achieved. However, friction arises between the archwire and bracket slot and decreases the retraction force applied to the anterior teeth. In clinical settings, because the amount of friction is unclear, and it is difficult to control net force acting on the teeth.

In another method, retraction springs are used to move the teeth, in which a force exerted by the spring acts directly on the teeth. This is known as a frictionless method, which is an advantage over the sliding mechanics. However, to achieve the desired movement pattern, an appropriate design of spring is necessary for producing a suitable force system. Various shapes of spring have already been developed [1,2,3,4,5,6,7,8,9,10,11]. Additionally, to move a tooth through a long distance by a single activation, it is necessary to reduce the spring constant (force-to-displacement ratio). For this purpose, a titanium molybdenum alloy (TMA) is used as a spring material, because its Young’s modulus is about one-third of that of stainless steel [8,9,10,11].

A new β-titanium alloy has been developed in Japan, which is named gum metal (GUM) [12]. This multi-functional titanium alloy has a low Young’s modulus, a high yield strain, and also good formability. These properties of GUM appear to be superior to those of TMA. Additionally, GUM wires have been used for improving the effectiveness of orthodontic treatments [13,14].

Since GUM is more expensive than TMA, it will be necessary to clear the advantages of using GUM. In this study, we evaluate the efficacy of a retraction spring made of GUM. Using double loop springs incorporated into an archwire, anterior teeth of maxillary dentition moved bodily for closing a retraction space. Long-term movements of the teeth were simulated by the finite element method (FEM). In comparison with the TMA spring, the features of the GUM spring are shown.

## 2. Materials and Methods

### 2.1. Finite Element Modle of Maxillary Dentition

**Figure 1:**
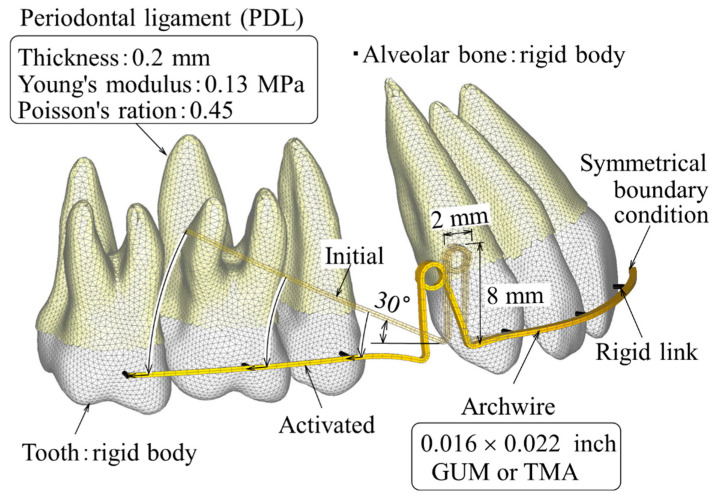
Finite element model for simulating extraction space closure using double loop spring.

Figure 1 shows a finite element (FE) model for simulating the extraction space closure using a double loop spring. Only the right side of the maxillary dentition was modeled because of its bilateral symmetry. A symmetrical boundary condition was applied to a median section of the archwire.

FE models of the teeth were made based on a dental study model (i21D-400C, Nissin Dental Products Inc., Kyoto, Japan) [15]. They were made by the following three steps. First, sectional images of the dental study model were taken using dental cone beam computed tomography (CBCT), AZ300CT (Asahi Roentgen, Co., Ltd., Kyoto, Japan). Second, using a 3D modeling software, 3D-Doctor (Able Software Corp., Lexington, MA, USA), a stereolithographic (STL) model of each tooth was constructed. Third, the surface of the STL model was meshed with shell elements using a meshing software, ANSYS AI*Environment (ANSYS Japan, Tokyo, Japan).

The long-term movement of the teeth was simulated based on the initial movement caused by elastic deformation of the periodontal ligament (PDL). To calculate the initial movement, the PDL with 0.2 mm thickness was constructed by extruding the root in the normal direction and meshing it with three-dimensional solid elements. The PDL was assumed to be a linear elastic material, whose Young’s modulus and Poisson’s ratio were 0.13 MPa and 0.45, respectively [16].

Young’s modulus of the teeth and alveolar bone (above 10 GPa) were much larger than that of the PDL, and thereby, the deformation of the teeth and alveolar bone could be neglected within the range of the orthodontic force. Hence, the teeth and alveolar bone were assumed to be rigid bodies. The rigid body of the teeth could be defined with the shell elements on the surfaces.

### 2.2. Finite Element Model of Archwire with Retraction Spring

Double loops of 2 mm diameter and 8 mm height were incorporated into an archwire of 0.016 × 0.022 inch. The posterior portion of the archwire was bent at a 30-degree angle, which was named the Gable bend. This bending was needed to prevent the lingual tipping of the anterior teeth and achieve bodily movement. This amount of Gable bend was selected as a typical one. The archwire was meshed with three-dimensional solid elements.

**Figure 2:**
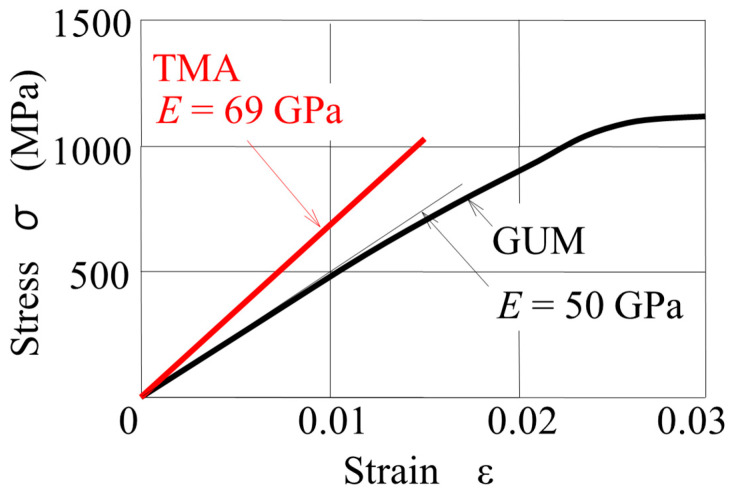
Stress–strain curves of GUM and TMA.

The archwire was made of TMA or GUM. Figure 2 shows the stress–strain curves of TMA and GUM [12]. TMA is a linear elastic material whose Young’s modulus is 69 GPa. GUM shows a nonlinear elasticity, in which the Young’s modulus shows a close to linear slope of 50 GPa which decreases with increasing strain. In the FE model, this nonlinear stress–strain relationship was approximated with a piecewise linear curve. The yield stresses of GUM and TMA were 1100 MPa and 1010 MPa, respectively [12,17].

### 2.3. Activation Method of Retraction Spring

In clinical settings, a wire is fixed firmly to the teeth through the brackets. Deformation of the bracket is negligible compared to the deflection of the archwire. In the FEM, the function of the bracket was modeled with a rigid link element. Using this element, the archwire was fixed directly to the anterior teeth at their bracket positions.

To activate the spring, firstly, the posterior portion of the archwire was rotated counterclockwise so that it was horizontal, then translated distally to the bracket positions of the molars. The amount of the translation was adjusted to produce a distal force of 2 N. This amount of force was selected by referring to an orthodontics textbook [18]. After the activation, the archwire was fixed to the posterior teeth with the rigid link elements.

### 2.4. Simulation Method of Long-Term Orthodontic Movement

When acting a force on a tooth, it moves due to the elastic deformation of the PDL. This is the initial movement. When maintaining the force, resorption and apposition of the alveolar bone occur at the compressive and tensile regions of the PDL, respectively. As a result of this bone remodeling, the alveolar socket moves. This is the orthodontic movement, which remains even after the force is removed. In the FEM, the movement of the alveolar socket was simulated. It was assumed that the alveolar socket moved in the same direction as the initial movement. This method has been developed in the previous articles [16,19].

The simulation procedure was constructed of two steps. First, the initial movement of each tooth was calculated. Second, the alveolar socket of each tooth was moved in the same direction as the initial movement. Repeating these two steps, the teeth moved by accumulating the initial movement. The force system acting on the teeth was updated at each step. The number of the repeating calculations, *N*, corresponded to an elapsed time. However, the *N* could not be converted to an actual time. A FE software, ANSYS19.1 (ANSYS Inc., Canonsburg, PA, USA), was used for the simulation.

When calculating the initial movement or displacing the alveolar socket, because the alveolar bone was assumed to be a rigid body, nodes of the outer surface of the PDL were fixed or displaced. Hence, the FE model of the alveolar bone was unnecessary to simulate the tooth movement.

## 3. Results

**Figure 3:**
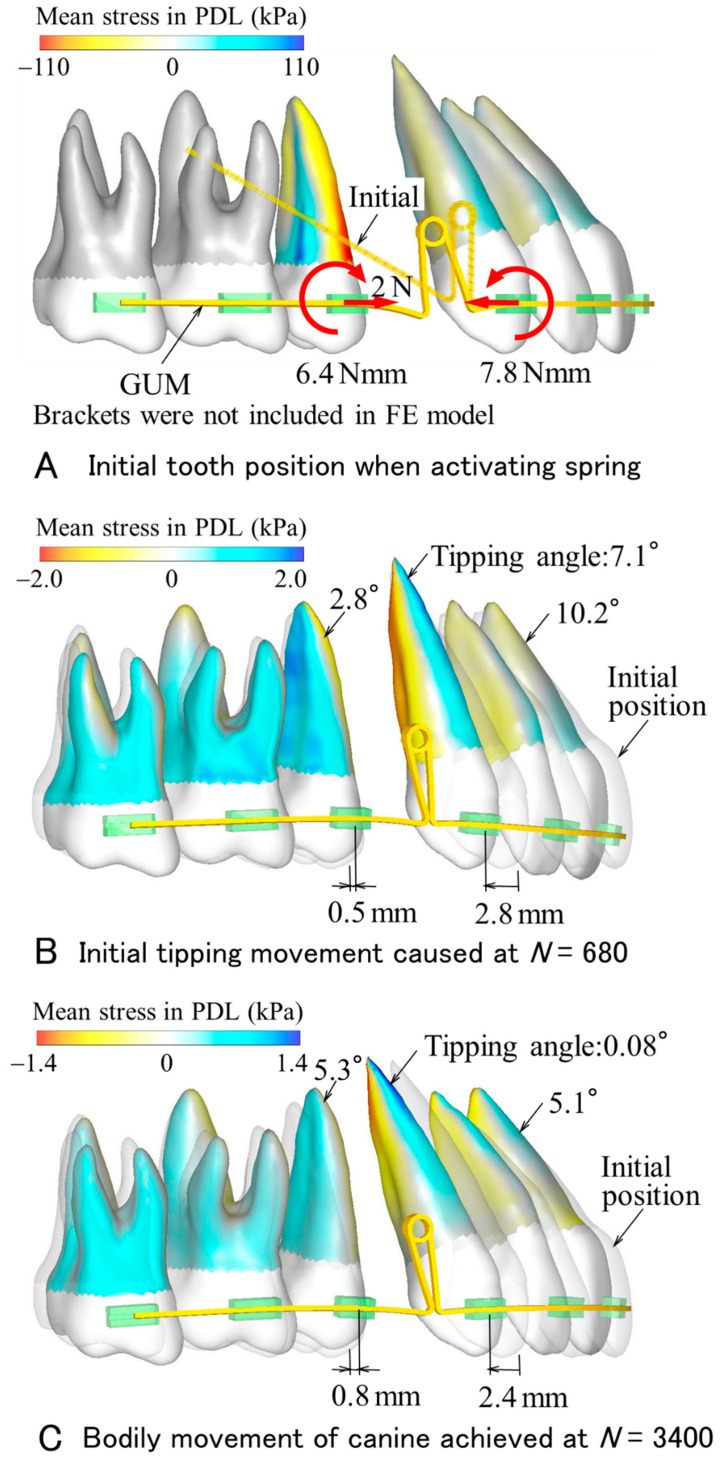
Tooth movement produced by GUM spring.

**Figure 4:**
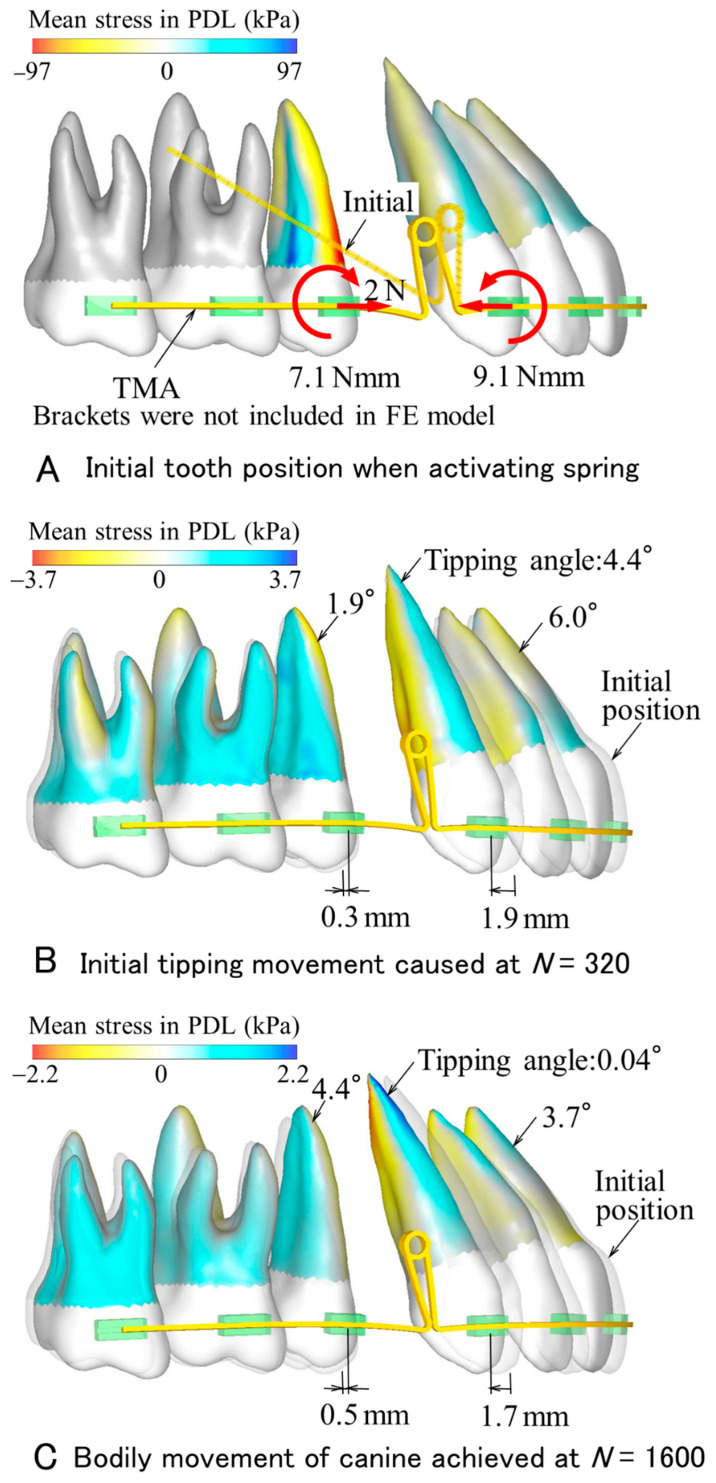
Tooth movement produced by TMA spring.

Figure 3 and Figure 4 show the tooth movement as calculated by the FE model and corresponding to the spring made of GUM (Figure 3) and made of TMA (Figure 4). In the case of the GUM spring, the distribution of the mean stress in the PDL is depicted on the roots with color contours. Although brackets were not included in the FE model, their dummies are illustrated to indicate bracket widths in clinical settings. At activation, forces of 2 N were applied to the second premolar and the anterior teeth. Moments acting on the second premolar and the canine were 6.4 and 7.8 Nmm, respectively. At the number of repeating calculations, *N* = 680, the anterior teeth tipped lingually. Tipping angles of the central incisor and canine were 10.2° and 7.1°, respectively. The second premolar tipped distally 2.8°. Bracket positions of the canine and second premolar moved distally 2.8 mm and mesially 0.5 mm. At this time, the amount of space closure was 2.8 + 0.5 = 3.3 mm. At *N* = 3400, although the central incisor tipped lingually 5.1°, the tipping angle of the canine became almost zero, 0.08°; namely, the canine moved bodily 2.4 mm. The second premolar moved 0.8 mm mesially and tipped distally 5.3°. When the repeating calculation continued further, the canine tipped mesially.

In the case of the TMA spring (Figure 3), the tooth movement process was similar to that in the GUM spring (Figure 4). The canine first tipped distally, then became upright. As a result of these movements, the canine moved bodily. At that time, in comparison with the GUM spring, the movement distance of the canine decreased to 1.7 mm, and also the number of repeating calculations required bodily movement decreased to *N* = 1600.

In the GUM and TMA springs, the maximum equivalent stresses produced in the archwires were 664 MPa and 898 MPa, respectively. These values were below the yield stresses of GUM (1100 MPa) and TMA (1010 MPa). Both springs were within their elastic ranges.

## 4. Discussion

### 4.1. Mechanism of Bodily Tooth Movement Produce by Retraction Spring

In both GUM and TMA springs, as the teeth moved, their movement pattern changed. The canine first tipped, then became upright. This was because the force system acting on the canine changed in the following way. At the activation of the spring, a moment acting on the canine was about 8 Nmm. Its moment-to-force (*M/F*) ratio was too small to move bodily, and thereby the anterior teeth tipped lingually. As the canine crown moved distally, since the retraction force decreased, the *M/F* ratio increased. When the *M/F* ratio rose above a sufficient amount for bodily movement, the anterior teeth began to become upright with a slight mesial movement. The tipping angle of the canine decreased and reached zero. At this time, the canine achieved bodily movement, while the central incisor still tipped due to an elastic deflection of the archwire. This unsteady movement process caused by retraction springs has already been clarified in the previous FEM simulations [16,20].

By the tipping and uprighting movement, a tooth can move bodily, even when the initial *M/F* ratio is below the necessary amount for bodily movement. When increasing the Gable bend in the archwire, the *M/F* ratio acting on the canine increases, and thereby the initial tipping angle decreases. The canine will become upright sooner, while a distance of the bodily movement will be reduced. If the *M/F* ratio reached a sufficient amount for bodily movement at activation, the tooth would only tip and not achieve bodily movement. This will not be the best way to increase the *M/F* ratio to be as large as possible.

### 4.2. Tooth Movement Efficacy of Gum Metal Spring

An amount of space closure is defined as the sum of distal movement of the canine and mesial movement of the second premolar. At the time when the canine moved bodily, the amount of space closure achieved by the GUM spring (3.3 mm) was 1.5 times of that by the TMA spring (2.2 mm). This is the biggest advantage of GUM spring. Assuming an extraction space to be 7 mm, it may be almost closed by twice the activations of the GUM spring.

In the GUM spring, the duration required for space closing will increase for the following reasons. Bodily movement of the canine was achieved by two types of movements, initial tipping, and subsequent uprighting. Due to the low Young’s modulus of GUM, the initial tipping angle and distal movement of the canine became larger, while a moment for uprighting the canine was smaller. These effects prolonged the duration needed for bodily movement. If the number of repeating calculations, *N*, is proportional to actual time, the ratio of the amount of space closure, δ, to *N* is proportional to the speed of the space closure. The δ/*N* was 3.3 mm/3400 = 0.97 μm in the GUM spring and 2.2 mm/1600 = 1.4 μm in the TMA spring. The time required for space closure with the GUM spring increases to 1.5 times that with the TMA spring. A rapid space closure may not be expected in the GUM spring.

At the time when the canine moved bodily, tipping angles of the central incisor and second molar were larger in the GUM spring (5.1° and 5.3°) than in the TMA spring (3.7° and 4.4°). This is due to the low Young’s modulus of GUM.

In the GUM spring, an amount of space closure increased, but its movement speed decreased. This is a typical property of retraction springs [16,20]. In general, a decrease in the stiffness of the retraction spring leads to an increase in movement distance, but a decrease in movement speed. Both increases in distance and the speed of bodily movement cannot be achieved at the same time. After considering these characteristics, an appropriate spring should be selected depending on the individual clinical case.

Compared to TMA, GUM shows a nonlinear elastic property within 2.5% strain, as shown in Figure 2. In the present GUM spring, the maximum equivalent stress generated was 664 MPa. In this range, GUM behaved almost as a linear elastic material. Hence, the nonlinear property did not affect the tooth movement efficacy, whose difference between the two springs was mainly due to their Young’s modulus. If the spring is designed so that the stress in the spring is larger, the nonlinear property of GUM may be exhibited in the tooth movement efficacy. Examining this effect is a future study.

### 4.3. Advantages and Limitations of Finite Element Simulation

An advantage of the present FEM was that it could simulate the long-term movement of the teeth. As the teeth moved, their movement pattern changed. Such a change was not predicted from the initial movement just after activation of the spring. Another advantage was that the efficacies of two different springs could be compared to each other under the same property of tooth movement. In clinical experiments, each patient has a different property of tooth movement, and thereby, the difference in movement pattern due to the springs may be unclear.

The PDL was assumed to be a linear elastic material, while the actual PDL has a non-linear elastic property. The validity of this assumption has been verified in the previous article, in which the tooth movement pattern caused by the retraction spring was almost independent of the non-linearity of the PDL [16].

It was assumed that the alveolar socket moved in the same direction as in the initial movement. This assumption is reasonable in the light of clinical experiences. For example, a lateral force acting on the crown causes the tipping movement of the tooth. In addition to the force, a moment is applied to the tooth for achieving bodily movement. This change in movement pattern occurs in the same way for both the initial and the orthodontic movements.

In clinical settings, various forces act on the teeth from the mandibular dentition, tongue, and cheeks, which may affect the tooth movement. These effects were neglected in the FEM simulation. The future study is to include such effects in the FE model and to simulate the tooth movement under conditions closer to clinical situations.

The present simulation results are for a typical FE model, which was constructed using a dental study model. When the shape and size of the teeth change, the quantitative results, such as the amount of tooth movement changes accordingly. This is a limitation of the FEM and should be noted when comparing the simulation results to those in clinical settings.

## 5. Conclusions

A double loop spring incorporated into the archwire was used to move anterior teeth distally to close an extraction space. This process was simulated by FEM. The teeth initially tipped and then became upright. As a result, the teeth moved bodily.

A spring made of gum metal (GUM), which had a low Young’s modulus, achieved a space closure of 3.3 mm. This amount was 1.5 times that achieved by a titanium molybdenum alloy (TMA) spring. However, the speed of space closure in the GUM spring was predicted to be smaller than that in the TMA spring.

## Data Availability

Data sharing is not applicable to this article.
